# Appetitive traits and long-term risk of disordered eating: a 3-year follow-up in children with overweight and obesity

**DOI:** 10.1007/s40519-026-01868-y

**Published:** 2026-05-20

**Authors:** Guro Dahle Christofersen, Dorthe Dalstrup Pauls, Jens Meldgaard Bruun

**Affiliations:** 1 Steno Diabetes Center Aarhus, Aarhus University Hospital, 8200 Aarhus, Denmark; 2 Department of Clinical Medicine, Aarhus University, 8200 Aarhus, Denmark; 3 Danish National Center for Obesity, 8200 Aarhus, Denmark

**Keywords:** Eating behavior, Childhood obesity, Lifestyle intervention, Pediatrics, CEBQ, EDE-Q

## Abstract

**Purpose:**

Disordered eating (DE) is common among children living with overweight and obesity, and individual appetitive traits may contribute to the persistence of both DE and excess weight. This study examined whether pre-intervention appetitive traits in 7- to 14-year-olds with overweight/obesity attending a 10-week lifestyle camp were associated with DE at a 3-year follow-up. Second, correlations between pre-intervention appetitive traits and Body Mass Index-Standard Deviation Score (BMI-SDS) 3 years later were explored.

**Methods:**

Children with overweight/obesity were recruited from two Danish lifestyle camps. Self-reported questionnaires were completed to collect data on appetitive traits and DE, assessed as overeating (OE) and loss-of-control (LOC) eating. Five categories (No DE, occasional/regular OE and occasional/regular binge eating (BE)) were generated based on OE frequency (1–3 vs. ≥4 episodes) and the presence or absence of LOC eating. Measured weight and height were used to calculate BMI-SDS.

**Results:**

190 children were included, and 102 reassessed at 3 years. Higher pre-intervention Food Responsiveness was associated with a twofold increased risk of Regular BE (vs. no DE) at follow-up (RRR = 2.05 95% CI: 1.03;4.09, *p* = 0.04). Higher scores of Slowness in Eating and Emotional Undereating were associated with Occasional OE 3 years later (RRR = 2.22 95% CI: 1.05;4.68, *p* = 0.04) and (RRR = 5.16 95% CI:1.80;14.79, *p* = 0.002), respectively. Pre-intervention Food Responsiveness correlated positively with BMI-SDS at 3 years, whereas Emotional Undereating correlated negatively (both *p* < 0.05).

**Conclusion:**

Identification of high-risk appetitive traits could offer a unique opportunity for early intervention to prevent later DE and weight gain in children with overweight/obesity.

*Level of evidence* Level II: evidence obtained from well-designed controlled trials without randomization.

*Trial registration* The study was preregistered at clinicaltrials.gov (ID: NCT04522921). The longitudinal part of this study was preregistered at OSF Registries (www.osf.io), https://doi.org/10.17605/OSF.IO/MPE4Z.

**Supplementary Information:**

The online version contains supplementary material available at 10.1007/s40519-026-01868-y.

## Introduction

Childhood overweight and obesity is an increasing problem worldwide [1]. Although the prevalence in Danish children is low it has increased over the last decades [2]. According to the World Health Organization (WHO), 390 million children were living with overweight or obesity in 2022 [3], and the World Obesity Federation expects this number to dramatically increase, reaching 770 million by 2035 [4]. This increment deserves public attention due to the higher risk of complications like hypertension, insulin resistance, and dyslipidemia in adulthood [5–7]. Childhood obesity also increases the risk of developing psychological and even psychiatric comorbidities [8]. Moreover, children living with overweight and obesity are at a particularly high risk of developing disordered eating (DE) [9, 10]. A meta-analysis has shown that more than a quarter of children with overweight and obesity report symptoms of DE, such as overeating (OE) and loss-of-control (LOC) eating [10]. Conversely, DE may contribute to excess weight gain and persistence of obesity [11, 12].

Moreover, longitudinal studies demonstrate that DE in childhood and adolescence is linked with adverse psychological outcomes, including depression, anxiety disorders, and eating-related psychopathologies such as binge eating disorders (BED), as well as metabolic syndrome later in life [12–15].

Studies exploring the effect of interventions on OE and LOC eating in children are limited. A 12-week randomized controlled trial comparing the effect of interpersonal psychotherapy (IPT), targeting interpersonal factors through psychoeducation, communication analysis and social skills training, with a standard health education (HE) in age 12–17, demonstrated that both interventions reduced LOC eating and limited expected BMI gain equally [16]; however, at the 1-year and 3-year follow-up, IPT showed greater efficacy in reducing LOC eating among ethnic-racial minority girls [17]. A similar pilot trial reported lower odds of LOC eating in the group receiving psychotherapy compared to family-based HE [18].

In a recent study from our group, Jakobsen et al. found a significant reduction in self-reported OE and LOC eating among children with overweight and obesity following a 10-week multicomponent lifestyle camp, with sustained improvements after 52 weeks [19]. However, in some studies [16–19], children continue to experience OE and LOC eating despite intervention efforts, indicating a possible presence of underlying factors that influence individual variability in response to different treatment approaches.

Appetitive traits reflect individual differences in response to internal and external food cues, thereby providing insight into when, why, and how we eat [20, 21]. Moreover, certain appetitive traits have been linked with symptoms of DE, including LOC eating [22]. In the present study, these traits were assessed using the Children’s Eating Behavior Questionnaire (CEBQ), a widely used and validated, parent-reported measure developed to capture key dimensions of children’s appetitive traits. The CEBQ was developed based on prior literature on eating behavior and obesity and further informed by parental reports of children’s eating styles. The CEBQ includes domains such as responsiveness to external food cues, emotional eating, satiety regulation and eating pace. Unlike earlier approaches conceptualizing eating styles categorically, the CEBQ measures appetitive traits along a continuum, capturing the degree to which each child displays specific eating behaviors [23]. These traits are commonly grouped into food approach behaviors (Food Responsiveness, Enjoyment of Food, Emotional Overeating, Desire to Drink) reflecting a greater drive to eat, and food avoidant behaviors (Satiety Responsiveness, Slowness in Eating, Emotional Undereating, Food Fussiness), reflecting greater sensitivity to internal satiety signals and reduced food intake [20].

According to the Behavioral Susceptibility Theory, children who are genetically predisposed to avid appetitive traits, including heightened Food Responsiveness and reduced Satiety Responsiveness, are more likely to overeat and develop obesity in obesogenic environments [24–26]. Cross-sectional studies support this theory, demonstrating that a higher food approach behavior is associated with overweight and obesity, while a higher food avoidant behavior is linked to underweight [27]. Furthermore, a 10-year longitudinal follow-up study by Derks et al. [28] demonstrated that high food approach behaviors in early childhood were linked to OE and LOC eating in adolescence.

To our knowledge, no previous study has examined the association between appetitive traits in children undergoing lifestyle interventions and the long-term risk of DE. Given ongoing concerns that lifestyle interventions may inadvertently contribute to the long-term development of DE in children [29, 30], examining these associations within a 3-year follow-up period may provide novel insight into whether appetitive traits represent stable risk factors for DE or are modifiable following lifestyle intervention.

The primary aim of this study was to investigate if pre-intervention appetitive traits were associated with DE, assessed as OE and LOC eating, 3 years after attending a lifestyle camp intervention in children with overweight and obesity. Additionally, the association between pre-intervention appetitive traits and long-term Body Mass Index-Standard Deviation Score (BMI-SDS) was investigated. We hypothesized that children reporting a higher food approach behavior (and lower food avoidance behavior) before the lifestyle intervention would have an increased risk of OE and LOC eating and exhibit a higher BMI-SDS at the 3-year follow-up.

## Methods

Children aged 7–14 years living with overweight or obesity were recruited for the present study as part of a prospective, nonrandomized controlled trial: The Childhood Obesity—Prevention of Diabetes Through Changed Eating Patterns Study (referred to as the COPE study) [31]. In brief, the COPE study aimed to investigate the effect of a higher protein diet in relation to weight loss in children living with overweight and obesity attending a 10-week lifestyle camp intervention. A referral to the camps required the presence of either overweight, obesity, loneliness, unhappiness, social- or family-related problems, as assessed by a general practitioner. The overarching goal of the camps was to improve the children’s physical health and quality of life through social activities, healthy meals, and daily exercise. The complete study design has been described previously [31]. No significant differences in changes in BMI-SDS were found between the intervention and control group (*p* = 0.24) after the 10 weeks, thus the groups were pooled in this study.

In the present study, all children attending either of two lifestyle camps (Julemærkehjem Hobro or Fjordmark) between October 2020 and March 2022 were invited to participate. All children who did not complete the Children’s Eating Behavior Questionnaire (CEBQ) at baseline, used to assess appetitive traits, were excluded. Furthermore, children with normal weight (BMI-SDS ≤ 1SD), a diagnosis of eating disorders or a disease requiring a special diet at baseline, were excluded. Other exclusion criteria were children or parents/guardians participating in another clinical trial as well as those who did not understand, were unwilling or unable to comply with the study protocol. Participating children were also required to have a written consent from parents/guardians to be part of the study.

### Questionnaires

Participants answered the CEBQ and selected items from the Eating Disorder Examination Questionnaire (EDE-Q). Questionnaires were delivered to parents/guardians electronically using REDcap.org database located at Aarhus University, Denmark.

### The Children’s Eating Behavior Questionnaire (CEBQ)

A Danish version of the CEBQ was developed and psychometrically validated in children with overweight and obesity, as described elsewhere [32]. The CEBQ is a 35-item parent-reported questionnaire containing eight different appetitive traits scales: Food Responsiveness, Emotional Overeating, Enjoyment of Food, Desire to Drink, Satiety Responsiveness, Slowness in Eating, Emotional Undereating and Food Fussiness [23]. Each item has a 5-option Likert response ranging from 1 = never to 5 = always. The eight distinct appetitive traits are scored from 1 to 5, with higher scores indicating a higher tendency of the specific behavior [23]. The CEBQ was answered by parents at baseline. According to a Cronbach’s alpha test, the internal reliability was generally within acceptable range (*α* ≥ 0.70). Slightly lower values were observed for Satiety Responsiveness (*α* = 0.65) and Emotional Undereating (*α* = 0.64).

### The Eating Disorder Examination Questionnaire (EDE-Q)

DE symptoms (OE/LOC eating) were evaluated using two modified questions from the adult Danish EDE-Q. The EDE-Q is a questionnaire based on the Eating Disorder Examination interview, which is recognized as the golden standard for assessing eating disorder psychopathology [33]. The following questions were used to asses OE and LOC eating at the 3-year follow-up: question number 13 (“Over the past 28 days, how many times have you eaten what other would regard as an unusually large amount of food (in relation to your size and age)?”) and 14 (“… On how many of these times did you have a sense of having lost control over your eating (at the time you were eating)?”). In accordance with the EDE-Q structure, participants were only presented to question 14, if answering ≥ 1 episode of OE to question 13 [19].

### Anthropometry

Body weight was measured using a bioelectric impedance (BIA) scale (InBody model 270, Hopkins medical Products, Grand Rapids, MI, USA), while height was measured using a fixed wall measuring tape. BIA was used as it has been demonstrated to effectively monitor longitudinal changes in body composition [34]. Anthropometry was assessed by camp staff. An age- and-sex-adjusted BMI-SDS was calculated using WHO AnthroPlus software. Children with BMI-SDS > 1SD were categorized as having overweight and children with a BMI-SDS > 2SD were categorized as having obesity [35].

### Statistics

In descriptive analyses, means ± standard deviations (SD) are presented for continuous data and absolute numbers and/or percentages [*n* (%)] for categorical data. All statistical analyses were performed using STATA/MP 18.5 (StataCorp LLC, USA). *P*-values < 0.05 were considered statistically significant.

Data on OE and LOC eating were categorized into five distinct categories: (1) No DE (No OE, no LOC), (2) Occasional OE (1–3 episodes OE, without LOC eating), (3) Occasional BE (1–3 episodes OE with LOC eating) (4) Regular OE (≥ 4 episodes OE, without LOC eating) and (5) Regular BE (≥ 4 episodes OE with LOC eating).

Multinomial logit models were used to examine associations between baseline appetitive traits and DE categories at the 3-year follow-up. No DE was set as the reference group. To explore a possible effect of the intervention, secondary multinomial logit analyses were conducted to investigate associations between baseline appetitive traits and DE following the 10-week intervention using the same modeling approach. Results are presented as relative risk ratios (RRR), 95% confidence intervals (95% CI) and p-values.

Scatterplot inspection revealed non-linear relationships between appetitive traits and BMI-SDS, why Spearman’s rank correlation was applied to examine associations between baseline appetitive traits and BMI-SDS at the 3-year follow-up. Additionally, corresponding analyses were performed to explore associations between appetitive traits at baseline and BMI-SDS following the 10-week intervention. Results from the Spearman’s rank analysis are presented as correlation coefficients (*r*_*s*_) with corresponding p-values. The correlation coefficients were interpreted according to cutoff values as follows; 0.10–0.29 as a weak correlation, 0.30–0.49 as a moderate correlation, and 0.50–1.0 as a strong correlation [36].

To identify potential differences between participants included and those lost to follow-up at 3 years, children with missing data on EDE-Q at the 3-year follow-up were compared to those with complete EDE-Q data on sex and baseline measures of DE, age, appetitive traits, and BMI-SDS. Dropout analyses were performed using unpaired t-tests for continuous data and Chi^2^ statistics for categorical data.

## Results

### Study population

**Fig. 1:**
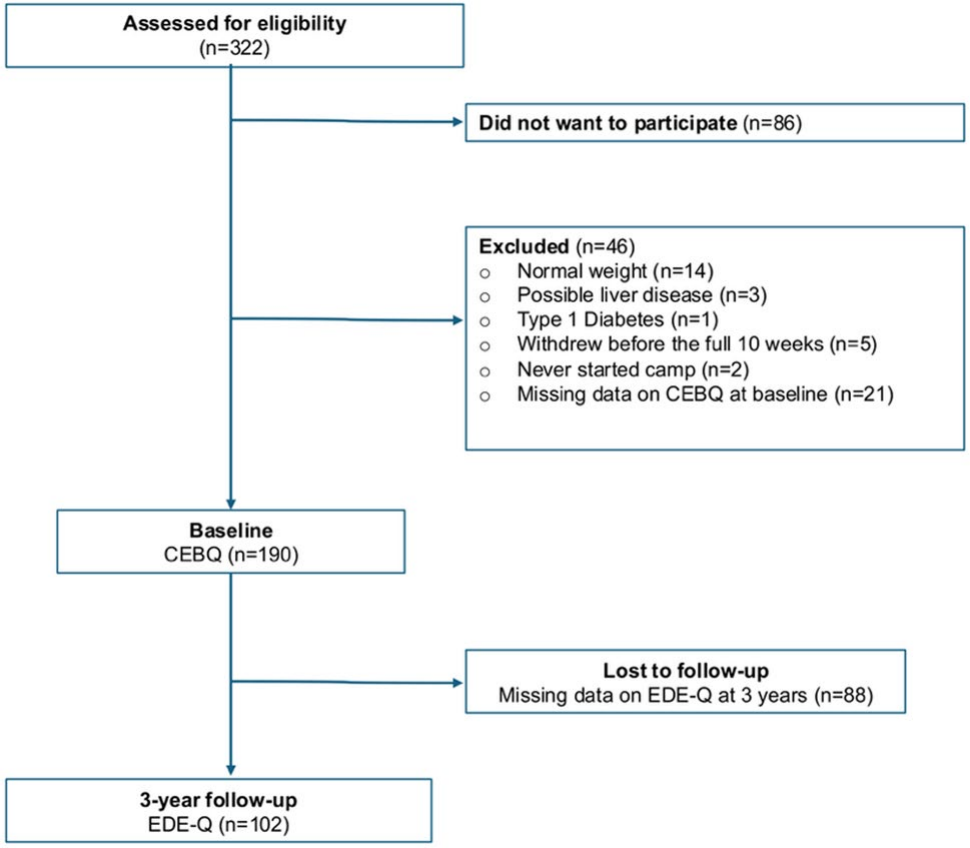
Flowchart of study participants. Abbreviations: CEBQ, Children’s Eating Behavior Questionnaire; EDE-Q, Eating Disorder Examination Questionnaire

In total, 322 children were invited to participate and 236 accepted the invitation. At baseline, 190 children answered the CEBQ, the primary exposure measure. Of those, 102 (54%) completed the EDE-Q at 3 years, which constituted the final endpoint (Fig. 1).

At baseline, participants had a mean age of 12.3 ± 1.4 years, 83% were living with obesity and 58% were girls. Baseline scores of food approach behaviors were generally high, with most participants scoring in the upper range (e.g., Food Responsiveness 3.49 ± 0.94, Enjoyment of Food 4.1 ± 0.62). Baseline scores of food avoidant behaviors were generally low, with most participants scoring in the lower range (e.g., Satiety Responsiveness 2.19 ± 0.62). (Supplementary 1).

**Fig. 2:**
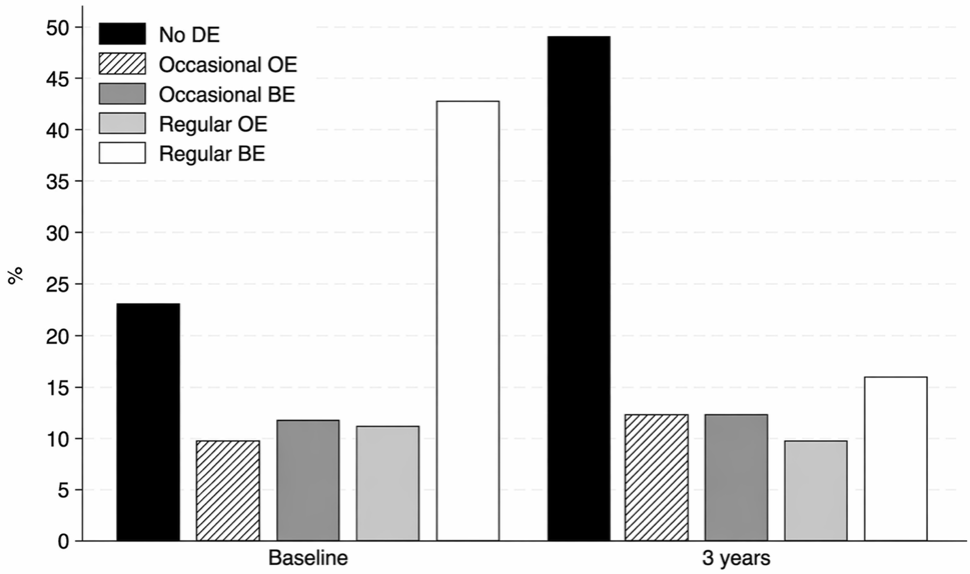
Distribution of DE categories at baseline and the 3-year follow-up. Abbreviations: DE, Disordered eating; OE, Overeating; BE, Binge eating

At the 3-year follow-up, participants had a mean age of 15.4 (± 1.3) and similar to baseline, 58% were girls. A subsample of participants had anthropometric measurements (*n*: 73) showing that the majority were living with obesity (55%). At the 3-year follow-up, 67% of the participants were attending primary school, while 17% were doing 10th grade at a voluntary Boarding school. Further, 13% of the participants reported Occasional BE, while 15% reported Regular BE at the 3-year follow-up (Table 1). Compared to baseline, the distribution of DE categories indicated a shift towards lower severity of DE at the 3-year follow-up, with an increased proportion reporting No DE (Fig. 2). Supplementary analyses examining changes from baseline to post-intervention (10 weeks) demonstrated a similar shift towards lower severity of DE (Supplementary 2). Additionally, reductions in several food approach behaviors were observed following the intervention (Supplementary 3).

**Table 1:** Participant characteristics at the 3-year follow-up (*n*: 102)

*Sex*	*n (%)*
Boys	43 (42%)
Girls	59 (58%)
	*Mean ± SD*
Age (years)	15.4 ± 1.3
*Daily activities*	*n (%)*
0–10th grade in community or private school	69 (67%)
10th grade at a voluntary boarding school	17 (17%)
Upper secondary education	5 (5%)
Vocational education	4 (4%)
Part-time work	2 (2%)
Full-time work	1 (1%)
My child doesn’t go to school or work	2 (2%)
Other	2 (2%)
*DE (OE with/without LOC)*^*a*^	*n (%)*
No OE, no LOC (No DE)	50 (49%)
1–3 OE without LOC (Occasional OE)	13 (13%)
1–3 OE with LOC (Occasional BE)	13 (13%)
≥ 4 OE without LOC (Regular OE)	10 (10%)
≥ 4 OE with LOC (Regular BE)	16 (15%)
*Anthropometry (n: 73)*	*Mean ± SD*
Weight (kg)	85.6 ± (15.6)
Height (meters)	1.71 ± (0.1)
BMI-SDS (WHO)^a^	2.1 ± (0.9)

^a^OE, Overeating; LOC, Loss-of-control; DE, Disordered eating; BE, Binge eating; BMI-SDS, Body Mass Index-Standard Deviation Score

In total, 88 (46%) participants were lost to the 3-year follow-up (Fig. 1). Drop-outs had a higher BMI-SDS at baseline compared to those with complete data (*p* < 0.01). No differences were observed in sex, baseline measures of age, DE or appetitive trait scores between participants with complete data on EDE-Q at 3 years and those lost to follow-up (data not shown).

### Associations between appetitive traits before camp and outcomes at the 3-year follow-up

Participants reporting higher scores of Food Responsiveness at baseline had a significantly higher risk of experiencing Regular BE at the 3-year follow-up compared to No DE (RRR = 2.05 95% CI: 1.03;4.09, *p* = 0.04). Among the food avoidant behaviors, both a higher pre-intervention Slowness in Eating and Emotional Undereating were associated with an increased risk of experiencing Occasional OE versus No DE at the 3-year follow-up, yielding RRRs of 2.22 (95% CI: 1.05;4.68, *p* = 0.04) and 5.16 (95% CI:1.80;14.79, *p* = 0.002), respectively (Table 2). In secondary analyses, no statistically significant associations were observed between any baseline appetitive trait and DE categories following the 10-week intervention.

**Table 2:** Associations between pre-intervention appetitive traits and DE at the 3-year follow-up

Appetitive traits^a^	No DE at 3 years follow-up	Occasional OE at 3 years follow-up	Occasional BE at 3 years follow-up	Regular OE at 3 years follow-up	Regular BE at 3 years follow-up
		RRR (95% CI), *p*-value	RRR (95% CI), *p*-value	RRR (95% CI), *p*-value	RRR (95% CI), *p*-value
FR_0_	Ref.	1.09 (0.54;2.18), *p* = 0.81	1.60 (0.78;3.26), *p* = 0.20	0.83 (0.38;1.81), *p* = 0.64	**2.05 (1.03;4.09), *p* = 0.04**
EOE_0_	Ref.	1.16 (0,59;2.29), *p* = 0.66	1.66 (0.83;3.30), *p* = 0.152	0.95 (0.45;2.01), *p* = 0.89	1.38 (0.73;2.58), *p* = 0.32
EF_0_	Ref.	1.23 (0.46;3.34), *p* = 0.68	1.01 (0.38;2.66), *p* = 0.99	0.62 (0.22;1.70), *p* = 0.35	1.58 (0.61;4.08), *p* = 0.35
DD_0_	Ref.	1.16 (0.61;2.20), *p* = 0.65	0.95 (0.49;1.84), *p* = 0.88	1.05 (0.51:2.17), *p* = 0.89	1.00 (0.55;1.83), *p* = 1.00
SR_0_	Ref.	1.86 (0.67;5.15), *p* = 0.23	0.67 (0.24;1.90), *p* = 0.46	0.98 (0.32;3.02), *p* = 0.97	0.47 (0.18;1.27), *p* = 0.14
SE_0_	Ref.	**2.22 (1.05;4.68), *p* = 0.04**	0.54 (0.24;1.20), *p* = 0.13	1.43 (0.64;3.19), *p* = 0.38	0.68 (0.33;1.38), *p* = 0.28
EUE_0_	Ref.	**5.16 (1.80;14.79), *p* = 0.002**	1.95 (0.81;4.66), *p* = 0.14	1.06 (0.42;2.63), *p* = 0.91	0.93 (0.44;1.98), *p* = 0.86
FF_0_	Ref.	0.59 (0.30;1.16), *p* = 0.13	1.45 (0.78;2.70), *p* = 0.25	1.04 (0,52;2.05), *p* = 0.92	1.02 (0.58;1.79), *p* = 0.95

Subscript 0 denotes baseline (pre-intervention)

^a^OE, Overeating; LOC, Loss-of-control; DE, Disordered Eating; BE, Binge eating; FR, Food Responsiveness; EOE, Emotional Overeating; EF, Enjoyment of Food; DD, Desire to Drink; SR, Satiety Responsiveness; SE, Slowness in Eating; EUE, Emotional Undereating; FF, Food Fussiness

A significant positive correlation was observed between pre-intervention Food Responsiveness and BMI-SDS at the 3-year follow-up (*r*_*s*_ = 0.26, *p* = 0.03), whereas Emotional Undereating, was negatively correlated with BMI-SDS (*r*_*s*_ = −0.24, *p* = 0.04) (Table 3). Pre-intervention Food Responsiveness and Desire to Drink were positively associated with BMI-SDS following the 10-week intervention (*r*_*s*_ = 0.20, *p* = 0.01 and *r*_*s*_ = 0.20, *p* = 0.01), while no significant associations were observed for the remaining traits.

**Table 3:** Associations between pre-intervention appetitive traits and BMI-SDS at the 3-year follow-up

Appetitive traits^a^	Correlation coefficient (*r*_*s*_), *p*-value
FR_0_	**0.26, *p* = 0.03**
EOE_0_	0.15, *p* = 0.19
EF_0_	0.17, *p* = 0.14
DD_0_	0.02, *p* = 0.86
SR_0_	− 0.15, *p* = 0.21
SE_0_	− 0.17, *p* = 0.16
EUE_0_	− **0.24, *p* = 0.04**
FF_0_	0.03, *p* = 0.77

Subscript 0 denotes baseline (pre-intervention)

^a^BMI-SDS, Body Mass Index-Standard Deviation Score; FR, Food Responsiveness; EOE, Emotional Overeating; EF, Enjoyment of Food; DD, Desire to Drink; SR, Satiety Responsiveness; SE, Slowness in Eating; EUE, Emotional Undereating; FF, Food Fussiness

## Discussion

This long-term intervention study is the first to demonstrate that higher pre-intervention Food Responsiveness is associated with an increased risk of reporting Regular BE three years later. Furthermore, higher pre-intervention scores of Emotional Undereating and Slowness in Eating were associated with an increased risk of reporting Occasional OE at the 3-year follow-up. In addition, higher pre-intervention Food Responsiveness was associated with a higher BMI-SDS at follow-up, whereas higher pre-intervention Emotional Undereating was associated with a lower BMI-SDS. Overall, an improvement in DE was observed from baseline to the 3-year follow-up.

The finding of heightened Food Responsiveness correlating with a longitudinally increased risk of experiencing Regular BE aligns with the limited research in this field [28, 37, 38]. This said, the present study included an intervention in children with overweight and obesity, who obtained a concomitant weight reduction, making the present study somewhat different from the previous research, primarily examining the longitudinal relationships between appetitive traits and DE, in cohorts including all weight classes [28, 37, 38].

Similar to our findings, a longitudinal study by Derks et al. found that greater scores of Food Responsiveness in early childhood (4–5 years) increase the odds of reporting DE symptoms (e.g., BE symptoms (OE/LOC or both), uncontrolled eating, and emotional eating) at age 12–14 years [28]. A cross-sectional secondary analysis found that LOC eating in 7–12 year olds correlated with higher scores of Food Responsiveness and Food Fussiness [37], while another study, using different tools to assess appetitive traits, show that certain early-life appetitive patterns (e.g., childhood OE) are associated with later manifestations of DE, including symptoms of BE [38]. Overall, this may indicate that early appetitive traits hold predictive value for the development of DE in later stages of life, emphasizing the potential value of early identification of high-risk appetitive traits, such as Food Responsiveness, in childhood, to enable early preventive strategies during critical developmental windows.

The present findings should also be interpreted considering the intervention context. As demonstrated in the supplementary analyses, the intervention reduced the prevalence of DE compared to baseline, possibly with long-term sustained improvements [19]. Similarly, reductions in several food approach behaviors were observed post-intervention. Notably, no associations were observed between pre-intervention appetitive traits and DE post-intervention, whereas some associations emerged at the 3-year follow-up. This pattern may suggest that the structured environment of the lifestyle camp intervention not only reduces the overall burden of DE, but may also temporarily attenuate or mask the influence of underlying appetitive traits on DE. As the children transition out of this structured setting and return to less regulated environments, individual differences in appetitive traits may re-emerge and contribute to the development of DE over time. This may explain why associations were not detectable in the short term, but some came evident at long-term follow-up.

Unexpectedly, higher scores on Emotional Undereating and Slowness in Eating, both considered food avoidant traits, were associated with an increased risk of reporting Occasional OE at follow-up. Contrary to our findings, previous studies have reported a negative relationship between Slowness in Eating and DE symptoms [28], while others have found a positive correlation between Emotional Undereating and avoidant/restrictive food intake disorder [39]. Given that all participants had overweight or obesity (BMI-SDS > 1SD) and potentially were referred to the camp due to psychosocial challenges, the present sample represents a population already predisposed to DE, as both excess weight and psychological distress have been linked with DE symptomatology [9, 10, 40, 41]. Further, 58% of our study population were girls, which are more susceptible to body dissatisfaction and DE than boys [42–44]. Given the limited sample size, sex-stratified analyses were not feasible.

In the present study, higher pre-intervention Food Responsiveness was positively associated with BMI-SDS at the 3-year follow-up, whereas higher pre-intervention Emotional Undereating was associated with a lower BMI-SDS. However, our findings also highlight important nuances. While Emotional Undereating was inversely associated with BMI-SDS at 3 years, this finding was not evident at 10 weeks, suggesting that it may not reflect a consistent relationship with weight development over time. In contrast, Food Responsiveness was positively associated with BMI-SDS both at the 10-week and 3-year follow-ups, indicating a more consistent relationship across time. This suggests that Food Responsiveness may present a more stable appetitive trait linked to long-term weight outcomes. These findings align with existing literature [27, 45], and support an association between appetitive traits and weight development in childhood. A 2021 meta-analysis reported that food approach traits (Food Responsiveness, Enjoyment of Food, Emotional Overeating, and Desire to Drink) in early childhood are prospectively associated with higher BMI-SDS over time, whereas food avoidant traits (Satiety Responsiveness, Slowness in Eating) are associated with lower BMI-SDS. Importantly, the same meta-analysis also suggested that higher adiposity measures may subsequently promote food approach behaviors and attenuate Satiety Responsiveness, indicating a potential bidirectional association [27]. Consistent with this, a Norwegian longitudinal study found that BMI predicted later appetitive traits, rather than the reverse. Specifically, increases in BMI between ages 6 and 12 were associated with subsequent increases in Food Responsiveness and Emotional Overeating across multiple time points, as well as higher Enjoyment of Food and lower Emotional Undereating during adolescence [45]. Thus, the relationship between appetitive traits and BMI appears complex, with no clear consensus regarding directionality, and may reflect reciprocal influences over time.

### Strengths and limitations

A major strength of this study is the long-term follow-up of children with overweight and obesity, a population at high-risk for developing DE. In addition, pre-intervention appetitive traits were assessed using a validated Danish version of the CEBQ, which has demonstrated satisfactory psychometric properties to assess appetitive traits in children with overweight and obesity [32].

Nevertheless, our results should be interpreted in light of certain limitations. At the time this study was initiated, a Danish version of the Youth EDE-Q (YEDE-Q) was not available or validated; therefore, two items from the adult EDE-Q [46], were adapted by the authors to assess OE/LOC in children. Since then, a Danish YEDE-Q translation has been developed and applied in adolescents with type 1 diabetes, showing similar phrasing [47]. However, further validation of the Danish YEDE-Q is warranted in community samples including children and adolescents with overweight and obesity. Consequently, the use of adapted, non-validated items may have introduced information bias, and some children may have had difficulties interpreting key concepts (e.g., “large amounts of food” or “a sense of loss of control”), potentially leading to an overestimation of the OE/LOC prevalence [46, 48]. If such misclassification was similar across exposure groups, associations would likely be biased towards the null; however, differential misclassification through systematic overreporting cannot be excluded. Additionally, although the CEBQ demonstrated acceptable internal reliability across most subscales, slightly lower Cronbach´s alpha values were observed for Satiety Responsiveness and Emotional Undereating in the present study. This may indicate reduced internal consistency within these subscales, which potentially could attenuate the observed associations. Moreover, LOC eating was assessed only among children reporting ≥ 1 episodes of OE over the past 28 days, but LOC eating can occur without OE. LOC eating, rather than OE, may be more critical in predicting future morbidity, including adiposity and psychiatric symptoms [49]. Therefore, our study may fail to capture children falling into this group, as well as other symptoms of DE. Finally, children who were lost to the 3-year follow-up exhibited higher baseline BMI-SDS values, suggesting that the observed correlations between baseline measures of Food Responsiveness/Emotional Undereating and BMI-SDS at three years may be stronger than reported. Nevertheless, the observed correlations were weak, and the findings should be interpreted with caution.

### What is already known on this subject?

- Overeating and loss-of-control eating is common among children with overweight and obesity and linked to adverse long-term outcomes.
- Appetitive traits have been associated with overeating and loss-of-control eating in pediatric cohorts including all weight classes, but whether pre-intervention appetitive traits predict long-term overeating with/without loss-of-control following lifestyle intervention is less explored.

### What this study adds?

- In children with overweight and obesity, higher scores on Food Responsiveness, Emotional Undereating and Slowness in Eating before a lifestyle intervention, increase the risk of experiencing overeating with/without loss-of-control eating. Food Responsiveness is also associated with a higher Body Mass Index-Standard Deviation Score three years later.
- These finding suggests that appetitive traits may serve as early markers to identify children with overweight/obesity at risk of persistent overeating with/without loss-of-control eating and unfavorable weight trajectories, thereby supporting early screening and targeted preventive strategies.

## Conclusion

In children with overweight and obesity referred to a 10-week multicomponent lifestyle camp, food approach behaviors were generally high before the intervention. Higher pre-intervention Food Responsiveness was associated with a greater risk of Regular BE and higher BMI-SDS at the 3-year follow-up. Moreover, higher pre-intervention Emotional Undereating and Slowness in Eating increased the risk of experiencing Occasional OE three years later. Within the context of a structured lifestyle intervention, these findings highlight that underlying appetitive traits remain relevant for long-term risk of DE and weight outcomes, although their influence may be attenuated in the short term. Thus, early identification of high-risk appetitive traits may provide predictive value for detecting children with overweight and obesity at risk of OE, BE and long-term weight gain, enabling targeted intervention strategies.

## Supplementary Information

Below is the link to the electronic supplementary material.

Supplementary file1 (DOCX 25 KB)

Supplementary file2 (DOCX 17 KB)

Supplementary file3 (DOCX 18 KB)

## Data Availability

Deidentified individual participant data will be made available upon reasonable request. Further enquiries can be directed to the corresponding author.

## Abbreviations

WHO: World Health Organization
DE: Disordered eating
OE: Overeating
LOC: Loss of control (eating)
BED: Binge eating disorder
IPT: Interpersonal psychotherapy
HE: Health education
BE: Binge eating
BMI-SDS: Body Mass Index-Standard Deviation Score
CEBQ: Children’s Eating Behavior Questionnaire
EDE-Q: Eating Disorder Examination Questionnaire
SD: Standard deviation
BIA: Bioelectric impedance
RRR: Relative risk ratio
95% CI: 95% Confidence intervals
YEDE-Q: Youth Eating Disorder Examination Questionnaire
