# Supplementary material for: Appetitive traits and long-term risk of disordered eating: a 3-year follow-up in children with overweight and obesity
Source: Eat Weight Disord. 2026 May 20;31(1):68. doi: 10.1007/s40519-026-01868-y (PMC13364941; doi:10.1007/s40519-026-01868-y)
Supplement: Supplementary file 1 — Supplementary file1 (DOCX 25 KB) [file 40519_2026_1868_MOESM1_ESM.docx]

**Supplementary 1.** Baseline characteristics (n: 190)

| **Sex** | *n (%)* |
| --- | --- |
| Boys | 80 (42%) |
| Girls | 110 (58%) |
|  | *Mean ± SD* |
| **Age (years)** | 12.3 ± 1.4 |
| **Appetitive traits^a^** | *Mean ± SD* |
| FR | 3.49 ± 0.94 |
| EOE | 2.78 ± 0.95 |
| EF | 4.1 ± 0.62 |
| DD | 2.79 ± 0.95 |
| SR | 2.19 ± 0.62 |
| SE | 2.34 ± 0.85 |
| EUE | 2.43 ± 0.74 |
| FF | 2.63 ± 0.98 |
| **DE (OE with/without LOC)^a^ *(n: 172)*** | *n (%)* |
| No OE, no LOC (No DE) | 40 (23%) |
| 1-3 OE without LOC (Occasional OE) | 17 (10%) |
| 1-3 OE with LOC (Occasional BE) | 21 (12%) |
| ≥ 4 OE without LOC (Regular OE) | 20 (12%) |
| ≥ 4 OE with LOC (Regular BE) | 74 (43%) |
| **Anthropometry *(n: 187)*** | Mean ± SD |
| Weight (kg) | 73.1 ± (15.8) |
| Height (meters) | 1.59 ± (0.1) |
| BMI-SDS (WHO)^a^ | 2.6 ± (0.7) |

^a^ FR (Food Responsiveness), EOE (Emotional Overeating), EF (Enjoyment of Food), DD (Desire to Drink), SR (Satiety Responsiveness), EUE (Emotional Undereating), FF (Food Fussiness), OE (Overeating), LOC (Loss-of-control), DE (Disordered Eating), BE (Binge eating), BMI-SDS (Body Mass Index Standard Deviation Score)
