# Supplementary material for: Appetitive traits and long-term risk of disordered eating: a 3-year follow-up in children with overweight and obesity
Source: Eat Weight Disord. 2026 May 20;31(1):68. doi: 10.1007/s40519-026-01868-y (PMC13364941; doi:10.1007/s40519-026-01868-y)
Supplement: Supplementary file 2 — Supplementary file2 (DOCX 17 KB) [file 40519_2026_1868_MOESM2_ESM.docx]

**Supplementary 2.** Changes in DE at baseline vs post-intervention (10-weeks)

| DE (OE with/without LOC) | Baseline (n: 172)  *n (%)* | Post-intervention (10-weeks) (n: 151)  *n (%)* | Changes from baseline to post intervention (10-weeks)  *RRR (95% CI), p-value* |
| --- | --- | --- | --- |
| No DE  (No OE, no LOC ) | 40 (23%) | 99 (66%) | *Ref.* |
| Occasional OE  (1-3 OE without LOC) | 17 (10%) | 15 (10%) | 0.35 (0.15;0.80), p=0.013 |
| Occasional BE  (1-3 OE with LOC) | 21 (12%) | 20 (13 %) | 0.35 (0.16;0.77), p=0.008 |
| Regular OE  (≥ 4 OE without LOC) | 20 (12 %) | 5 (3%) | 0.09 (0.03;0.27), p < 0.001 |
| Regular BE  (≥ 4 OE with LOC) | 74 (43 %) | 12 (8%) | 0.018 (0.01;0.06), p < 0.001 |

DE (Disordered Eating), OE (Overeating), LOC (Loss-of-control), BE (Binge eating)

Estimates obtained from multinomial logistic regression models.
