# Supplementary material for: Appetitive traits and long-term risk of disordered eating: a 3-year follow-up in children with overweight and obesity
Source: Eat Weight Disord. 2026 May 20;31(1):68. doi: 10.1007/s40519-026-01868-y (PMC13364941; doi:10.1007/s40519-026-01868-y)
Supplement: Supplementary file 3 — Supplementary file3 (DOCX 18 KB) [file 40519_2026_1868_MOESM3_ESM.docx]

**Supplementary 3.** Changes in appetitive traits at baseline vs post-intervention (10-weeks)

| Appetitive traits^a^ | Baseline  *Mean ± SD* | Post-intervention (10-weeks)  *Mean ± SD* | Change  *β, p-value* |
| --- | --- | --- | --- |
| FR | 3.49 ± (0.94) | 2.67 ± (0.94) | - 0.84, p < 0.001 |
| EOE | 2.78 ± (0.95) | 2.24 ± (0.81) | - 0.56, p < 0.001 |
| EF | 4.10 ± (0.62) | 3.99 ± (0.62) | - 0.15, p=0.004 |
| DD | 2.79 ± (0.95) | 2.54 ± (0.86) | - 0.24, p=0.003 |
| SR | 2.19 ± (0.62) | 2.64 ± (0.61) | + 0.45, p < 0.001 |
| SE | 2.34 ± (0.85) | 2.68 ± (0.73) | + 0.34, p < 0.001 |
| EUE | 2.43 ± (0.74) | 2.37 ± (0.73) | - 0.07, p=0.250 |
| FF | 2.63 ± (0.98) | 2.16 ± (0.77) | - 0.42, p < 0.001 |

^a^ FR (Food Responsiveness), EOE (Emotional Overeating), EF (Enjoyment of Food), DD (Desire to Drink), SR (Satiety Responsiveness), EUE (Emotional Undereating), FF (Food Fussiness)

Estimates obtained from linear mixed-effects models.
